# Serum 25-Hydroxyvitamin D and the risk of mortality in adult patients with Sepsis: a meta-analysis

**DOI:** 10.1186/s12879-020-4879-1

**Published:** 2020-03-04

**Authors:** Yuye Li, Shifang Ding

**Affiliations:** 1 Department of Pulmonary and Critical Care Medicine, Shandong Provincial Chest Hospital, Shandong University, Jinan, 250002 China; 2 Division of Intensive Care Unit, Qilu Hospital, Shandong University, No. 107, Wenhua West Road 107, Lixia District, Jinan, 250002 China

**Keywords:** Vitamin D, 25-hydroxyvitamin D, Mortality, Sepsis, Meta-analysis

## Abstract

**Background:**

Vitamin D deficiency has been related to the risk of sepsis. However, previous studies showed inconsistent results regarding the association between serum 25-hydroxyvitamin D (25 (OH) D) and mortality risk in septic patients. We aimed to evaluate the relationship between serum 25 (OH) D at admission and mortality risk in adult patients in a meta-analysis.

**Methods:**

Follow-up studies that provided data of multivariate adjusted relative risk (RR) between serum 25 (OH) D and mortality risk in septic patients were retrieved via systematic search of PubMed and Embase databases. A random effect model was used to pool the results.

**Results:**

Eight studies with 1736 patients were included. Results of overall meta-analysis showed that lower 25 (OH) D at admission was independently associated with increased risk or mortality (adjusted RR: 1.93, *p* < 0.001; I^2^ = 63%) in patients with sepsis. Exploring subgroup association showed that patients with severe vitamin D deficiency (25 (OH) D < 10 ng/ml) was significantly associated with higher mortality risk (adjusted RR: 1.92, *p* < 0.001), but the associations were not significant for vitamin D insufficiency (25 (OH) D 20~30 ng/ml) or deficiency (25 (OH) D 10~20 ng/ml). Further analyses showed that the association between lower serum 25 (OH) D and higher mortality risk were consistent in studies applied different diagnostic criteria for sepsis (systemic inflammatory response syndrome, Sepsis-2.0, or Sepsis-3.0), short-term (within 1 month) and long-term studies (3~12 months), and in prospective and retrospective studies.

**Conclusions:**

Severe vitamin D deficiency may be independently associated with increased mortality in adult patients with sepsis. Large-scale prospective studies are needed to validate our findings.

## Background

Sepsis is a common comorbidity in critically ill patients [1, 2]. The incidence of sepsis in critically ill patients remains high, probably due to the aging of the global population, increased usages of the invasive monitoring and treatment, emergence of the antibiotic resistance, and growing application of immunosuppressants etc. [2–4]. The mortality of patients with sepsis is very high, which is reported to be more than 30% according to previous studies [5]. For those with septic shock or multiple organ failure, the mortality could be more than 90% [5]. The pathogenesis of sepsis is rather complicated, and accordingly, treatments for sepsis are clinically difficult [5–7]. Therefore, identification of risk factors which are associated with poor prognosis in patients with sepsis is important for improvement of risk stratification and development of novel treatment target for the disease.

Vitamin D is a group of steroid hormones which mediate many physiological processes, including bone metabolism [8], calcium homeostasis [9], extraskeletal metabolism [10], cardiovascular homeostasis [11], and more importantly immune functions [12]. Pathophysiologically, vitamin D deficiency has been related to inflammation and immune dysfunction [13, 14], which may be the potential reason for the increased susceptibility of the individual to severe infection or sepsis. Previous studies showed that vitamin D deficiency, defined as lower than normal serum 25-hydroxyvitamin D (25 (OH) D), is independently associated with higher incidence of sepsis in critically ill patients [15–18]. However, it remains unknown whether lower serum 25 (OH) D predicts poor prognosis in patients with sepsis. Although accumulating studies have been published to evaluate the association between vitamin D deficiency and mortality risk in septic patients [19–26], the results were inconsistent. A previous meta-analysis including five observational studies showed that vitamin D deficiency was not associated with mortality risk in patients with sepsis [17]. However, since this meta-analysis only included studies with univariate analysis for the association between vitamin D and mortality, the results may be confounded [17]. Moreover, one qualified study [19] was missing from the previous meta-analysis, and some recently published studies were not included [23–26]. Therefore, we aimed to perform an updated meta-analysis to comprehensively evaluate the association between serum 25(OH) D and mortality risk in adult patients with sepsis.

## Methods

We followed the Preferred Reporting Items for Systematic Reviews and Meta-analyses (PRISMA) statement [27] and the Cochrane’s Handbook [28] during the designing, performing, and presenting the results of the meta-analysis.

### Database search

Using a combined database search terms, the electronic databases of PubMed and Embase databases were searched for studies evaluating the association between vitamin D deficiency and mortality in patients with sepsis. The search terms included: (1) “vitamin d” OR “25-hydroxyvitamin D” OR “25 (OH) D” OR “1,25-dihydroxyvitamin D” OR “1,25 (OH) D” OR “vitamin d2” OR “vitamin d3” OR “ergocalciferol” OR “cholecalciferol” OR “calcidiol” OR “calcifediol” OR “calcitriol” and (2) “sepsis” OR “septicemia” OR “septic”. We limited the search to human studies published in English or Chinese. To further identify additional studies, a manual search of the references of related articles or reviews was also performed as a complementation. The final literature search was performed on September 14, 2019. The full search strategy for PubMed is listed in Supplementary file 1.

### Study selection

A study was included into the meta-analysis if they met all of the following criteria: 1) designed as a longitudinal follow-up study; 2) included adult patients with sepsis; 3) serum 25 (OH) D was measured at admission and accordingly defined patients with vitamin D deficiency at baseline as exposure; 4) reported all-cause mortality during follow-up in septic patients with and without vitamin D deficiency at baseline; and 5) reported relative risk of all-cause mortality in septic patients with vitamin D deficiency compared to those without vitamin D deficiency after controlling for potential confounding factors. Reviews, cross-sectional studies, preclinical studies, duplications, and studies irrelevant to the aim of the meta-analysis were excluded. For studies of the same cohort with different follow-up durations, those with the longest follow-up durations were included.

### Data extraction and quality evaluation

The processes of literature search, data extraction, and quality evaluation were performed by two independent authors separately. When disagreements occurred, two authors discussed to reach a consensus. We extracted data regarding the study (authors, publication year, country, design, diagnostic criteria for sepsis and clinical setting), patients (sample size, age, and sex), exposure (timing, method, and cutoff value for 25 (OH) D), and outcome (follow-up duration, outcome reported, and confounding factors adjusted). Risk ratio (RR) and corresponding 95% confidence intervals (CI) after adequate controlling of the confounding factors were extracted or calculated to indicate the risk of all-cause mortality in septic patients with vitamin D deficiency compared to those without vitamin D deficiency. The definitions of vitamin D insufficiency, deficiency, or severe deficiency were in accordance with those applied in the original studies. We applied the Newcastle-Ottawa Scale (NOS) to evaluate the quality of the included study [29]. The criteria of NOS mainly included three domains: selection of the study groups; comparability of the groups; and ascertainment of the outcome of interest [29].

### Statistical analyses

As indicated by the Cochrane’s Handbook, RRs and their stand errors (SEs) were logarithmically transformed to be pooled in the meta-analysis to stabilize variance and normalized the distribution [28]. A Cochrane’s Q test was used for evaluate the heterogeneity of the meta-analysis. Moreover, we also calculated I^2^ statistic to reflect the extent of heterogeneity. A significant heterogeneity was deemed if I^2^ > 50% [30]. We used the random-effect model to combine the results because this model has been considered to incorporate the heterogeneity of the included studies thereby leading to a more generalized result [28]. We performed a sensitivity analysis, which exclude one study at a time to evaluate the robustness of the study [31]. Subgroup analyses according to the extent of vitamin D deficiency, diagnostic criteria for sepsis, follow-up durations, and design of the studies were also performed to evaluate the impacts of these features on the main results. Since the cut-off values for vitamin D insufficiency, deficiency, or severe deficiency were not established in patients with sepsis, the definitions of US Endocrine Society clinical practice guidelines were used [32]. Accordingly, optimal vitamin D level, vitamin D insufficiency, and deficiency are defined as serum 25 (OH) D > 30 ng/ml, 21–29 ng/ml, and < 20 ng/ml respectively [32]. Funnel plots were constructed, the symmetry of which could indicate the potential publication bias of the meta-analysis. A further analysis with the Egger’s regression test was also performed to evaluate the publication bias [33]. All of these statistical analyses were performed with RevMan (Version 5.1; Cochrane Collaboration, Oxford, UK) and STATA software (Version 12.0; Stata Corporation, College Station, TX).

## Results

### Literature search

**Fig. 1:**
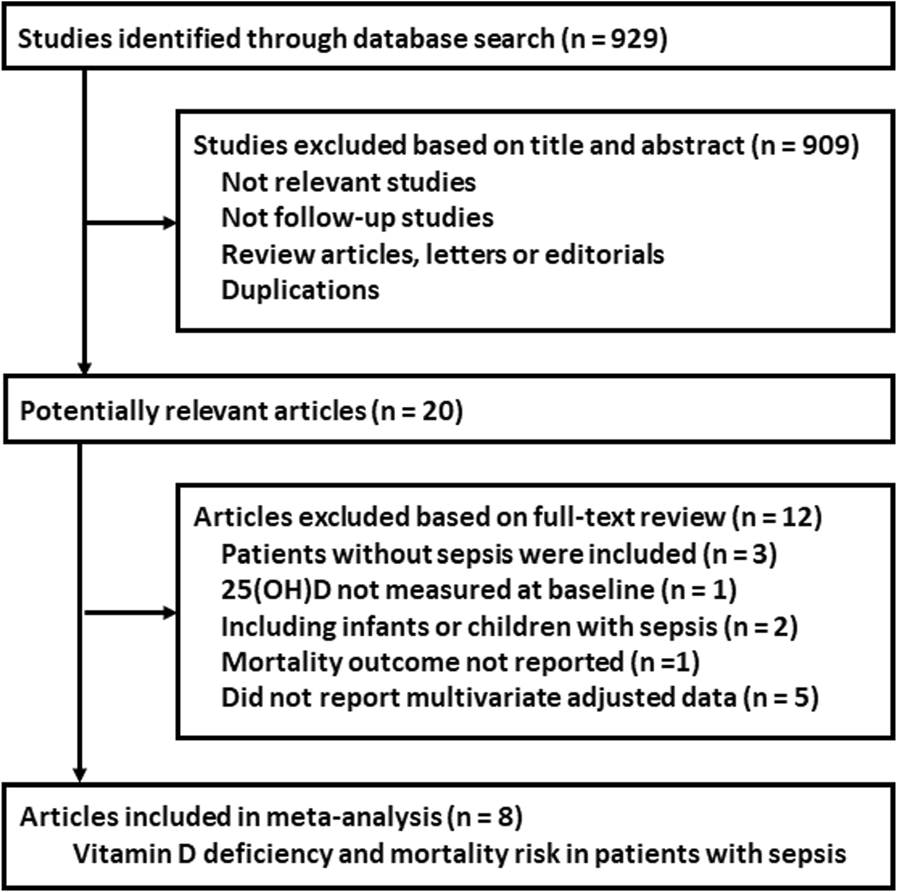
Flowchart of database search and study identification

The process of literature search and study identification was summarized in Fig. 1. Briefly, 929 studies were retrieved by initial database search. By screening via titles and abstracts, 909 studies were subsequently excluded, mainly because they were irrelevant to the objective of the meta-analysis. The remaining 20 studies underwent full-text review, of which 12 studies were further excluded because three did not included patients with sepsis, one did not measure serum 25(OH) D at baseline, two included infants or children with sepsis, one did not report mortality outcome, and the other five did not report multivariate adjusted RRs. Finally, eight follow-up studies were included in this meta-analysis [19–26].

### Study characteristics and quality evaluation

The characteristics of the included studies were listed in Table 1. Overall, this meta-analysis included eight follow-up studies with 1736 septic patients [19–26], among which, four were prospective [21, 24–26] and the other four were retrospective [19, 20, 22, 23]. Three of the studies were performed in the US [19, 20, 22], three in Europe [21, 25, 26], and the other two in Asia [23, 24]. All of the included studies were performed in the intensive care units (ICU) except for one study, which was performed in the emergency department (ED) [24]. Systemic inflammatory response syndrome (SIRS) was used as the diagnostic criteria for sepsis in three studies [19–21], while three studies used Sepsis-2.0 criteria [22–24], and the other two used Sepsis-3.0 criteria [25, 26]. The sample sizes of the included studies varied from 57 to 610. The mean ages of the patients included in each study ranged from 57 to 75 years, and the proportions of male patients varied from 43 to 71%. Serum 25 (OH) D was measured at admission for all of the included studies. Six of them used chemiluminescence immunoassay [19, 20, 22–24, 26], one used enzymatic immunoassay [21], while the other one did not describe the method of serum 25 (OH) D measuring [25]. All of the included studies analyzed serum 25 (OH) D as categorized variables. According to the definitions of the US Endocrine Society clinical practice guidelines on severity of vitamin D deficiency, one study provided mortality data for patients with vitamin D insufficiency, deficiency, and severe deficiency separately, and the other study provided data for patients with vitamin D deficiency and severe deficiency separately. These datasets were included independently. The follow-up durations varied from within hospitalization to 12 months. Established scores for risk stratification in sepsis patients in ICU or ED were adjusted for all of the included studies when presenting the association between serum 25 (OH) D and mortality risk, including the Score on the Acute Physiology and Chronic Health Evaluation II, the Simplified Acute Physiology Score, the Sequential Organ Failure Assessment Score, and the Mortality in the Emergency Department Sepsis Score. The quality of the included studies was moderate, with NOS varying from 6 to 8 stars.

**Table 1:** Characteristics of the included studies

Study	Design	Country	Diagnosis of Sepsis	Clinical setting	Sample size	Measured timing for 25 (OH) D	Measuring methods for 25 (OH) D	Mean age	Male	Cut-off for 25 (OH) D	Outcome data	Variables adjusted	NOS
								years	%	pg/ml			
Rech 2014 [20]	Retrospective	the US	SIRS	ICU	121	At admission	CLIA	65.1	48.8	< 15	Mortality at 1 month	Age, African American race, APACHE II score, pulmonary source of sepsis, and any vitamin D supplementation	6
Moromizato 2014 [19]	Retrospective	the US	SIRS	ICU	568	At admission	CLIA	65.9	43.8	< 30	Mortality during hospitalization, at 1 month, at 3 months	Age, gender, race, type (surgical vs medical), and APACHE II score	7
Ala-Kokko 2016 [21]	Prospective	Finland	SIRS	ICU	610	At admission	EIA	62.4	63	< 10, 10–20, 20–30	Mortality at 3 months	APACHE II score, multiorgan failureduring 0–4 days, DM, use of systemic corticosteroids, LOH before ICU admission and AKI	8
De Pascale 2016 [22]	Retrospective	the US	Sepsis-2.0	ICU	107	At admission	CLIA	65.7	57.9	< 7	Mortality at 12 months	Age, history of CKD, SAPS II, SOFA, and ARDS	6
Trongtrakul 2017 [24]	Prospective	Thailand	Sepsis-2.0	ED	110	At admission	CLIA	57.9	49.1	< 12, 12–20	Mortality at 1 month	Age, sex and APACHE II score	7
Ding 2017 [23]	Retrospective	China	Sepsis-2.0	ICU	57	At admission	CLIA	57.4	57.9	< 20	Mortality at 1 month	Age, sex, APACHE II score, and SOFA	6
Suberviola 2019 [26]	Prospective	Spain	Sepsis-3.0	ICU	75	At admission	CLIA	64	70.7	< 10	Mortality during hospitalization	Age, sex, immunosuppression status, Charlson index, APACHE II score, and hepatic failure	6
Mirijello 2019 [25]	Prospective	Italy	Sepsis-3.0	ICU	88	At admission	NA	75	57.9	< 7	Mortality at 1 month, and at 3 months	Age, sex, and MEDS score	7

*ED* Emergency department, *ICU* Intensive care unit, *CLIA* Chemiluminescence immunoassay; *EIA* Enzymatic immunoassay, *NA* Not available, *APACHE II score* Score on the Acute Physiology and Chronic Health Evaluation II, *SAPS* Simplified Acute Physiology Score, *SOFA* Sequential Organ Failure Assessment, *MEDS* Mortality in the Emergency Department Sepsis, *DM* Diabetes mellitus, *LOH* Length of hospitalization, *AKI* Acute kidney injury, *CKD* Chronic kidney disease, *ARDS* Acute respiratory distress syndrome, *SIRS* Systemic inflammatory response syndrome

### Association between serum 25 (OH) D and mortality risk in patients with sepsis

**Fig. 2:**
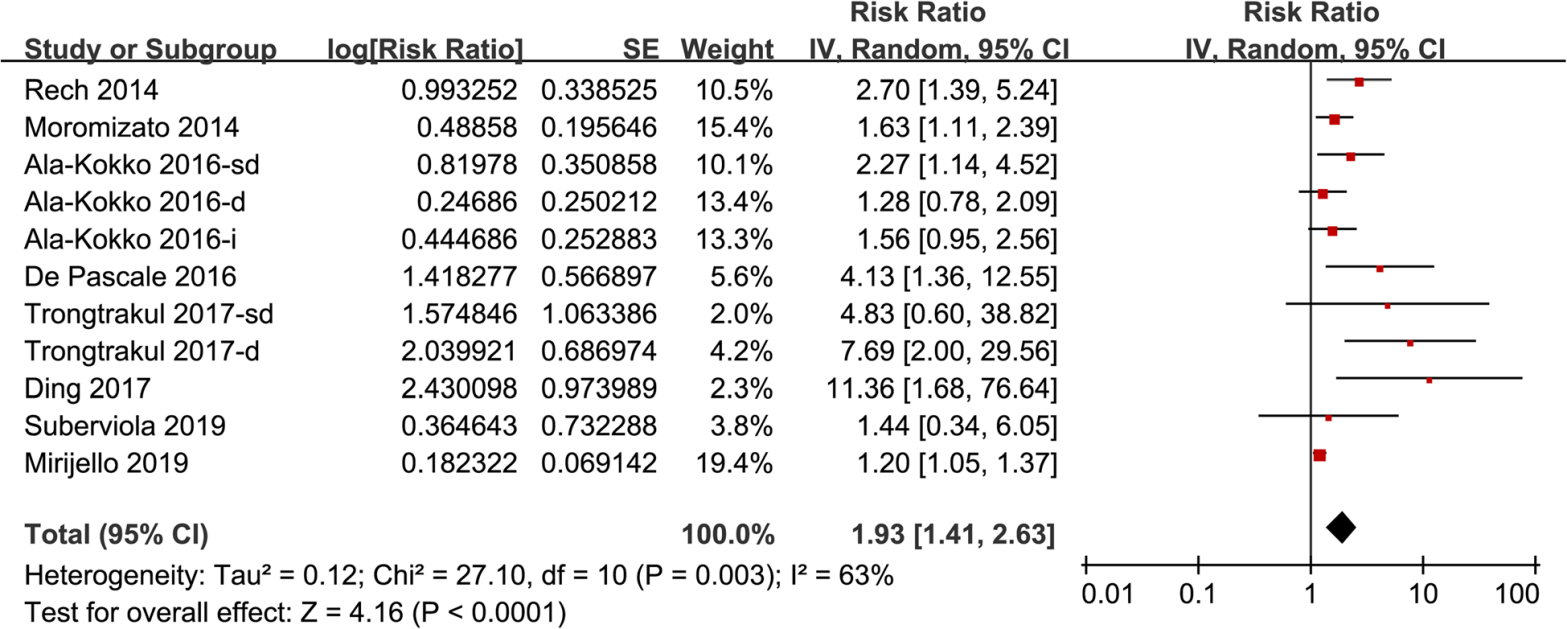
Forrest plots for the association between serum 25 (OH) D and mortality risk in patients with sepsis: results of the main meta-analysis

**Fig. 3:**
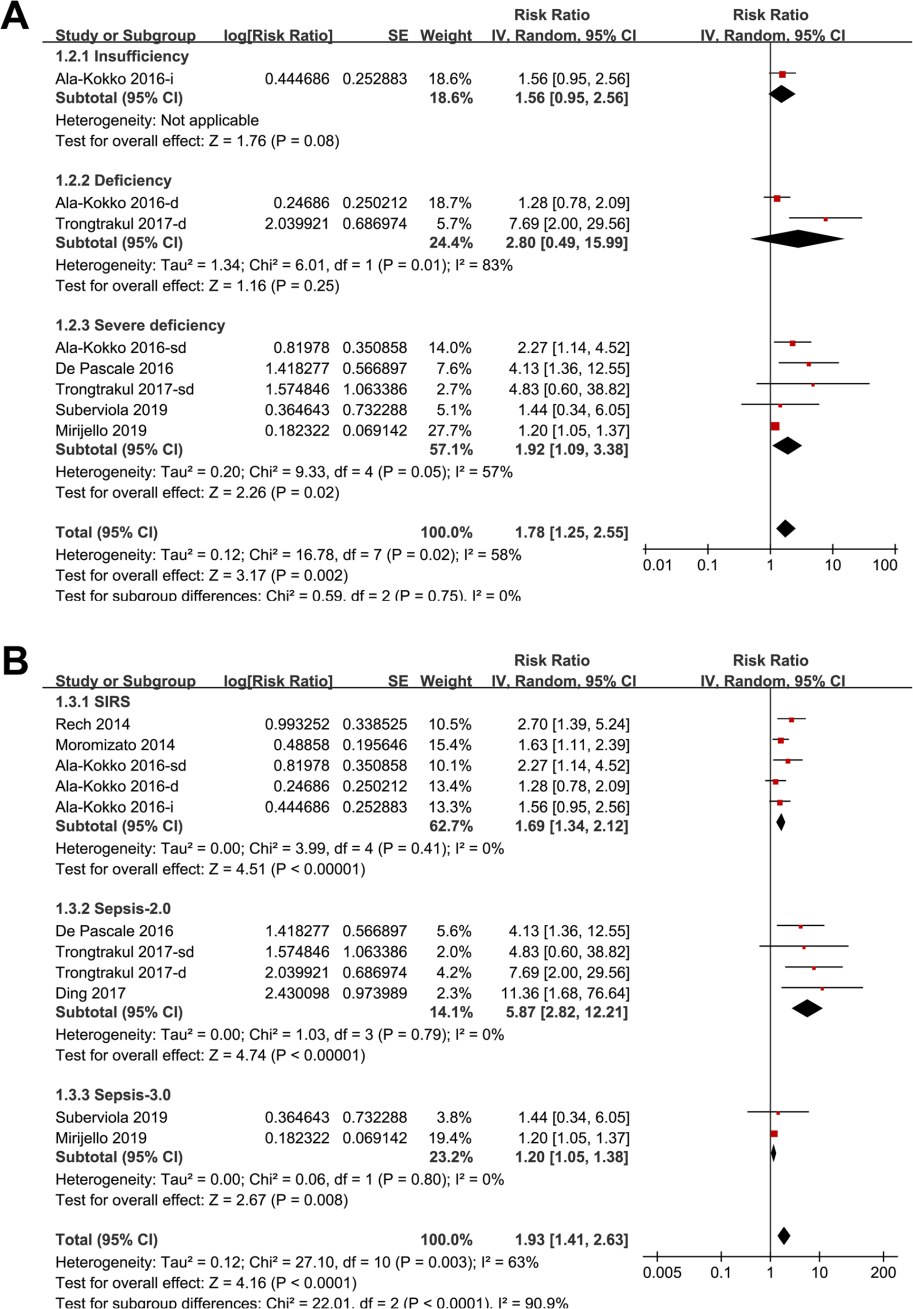
Subgroup analysis for the association between serum 25 (OH) D and mortality risk in patients with sepsis; **a** subgroup analysis according to the degree of vitamin D deficiency; **b** subgroup analysis according to the diagnostic criteria for sepsis

**Fig. 4:**
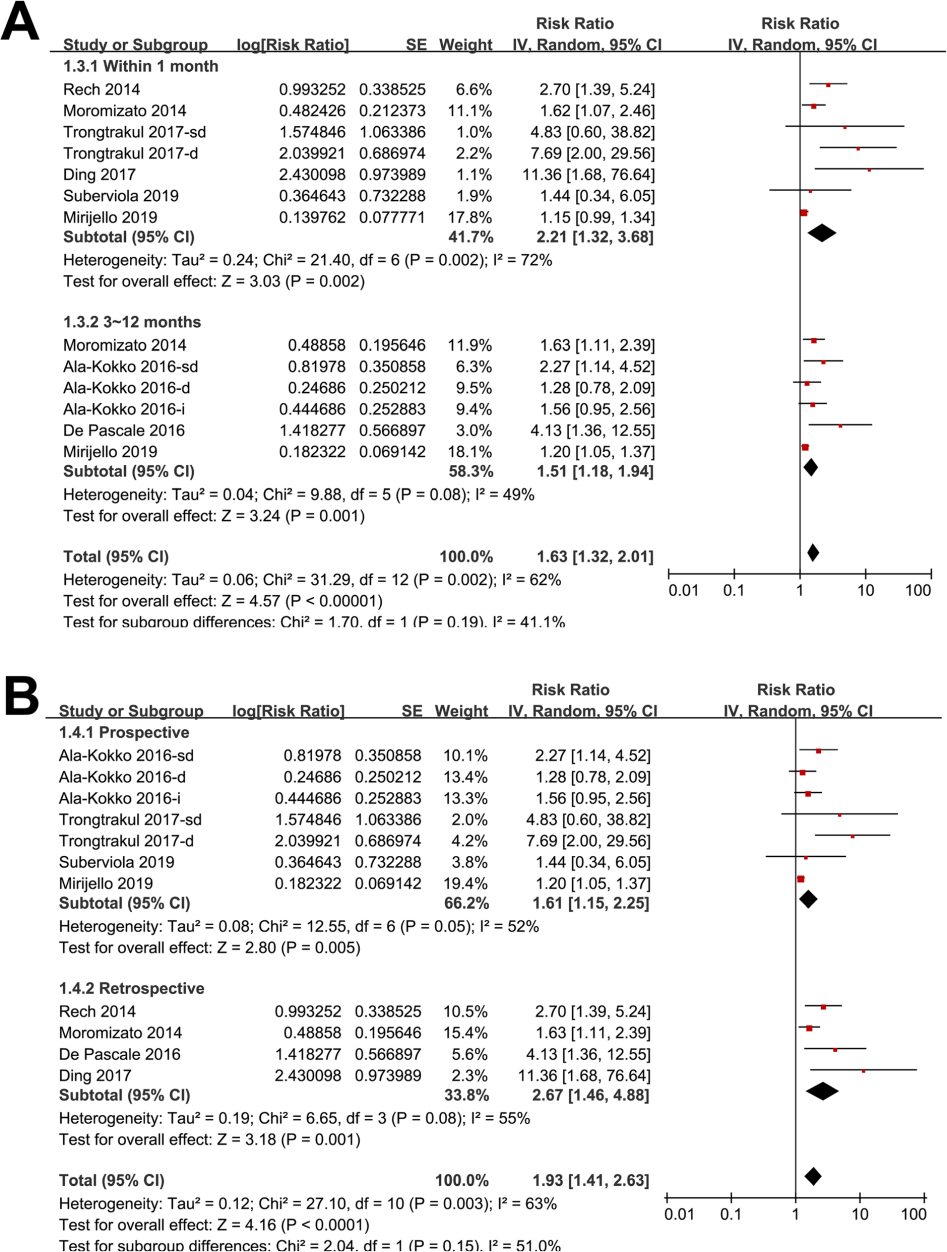
Subgroup analysis for the association between serum 25 (OH) D and mortality risk in patients with sepsis; **a** subgroup analysis according to the follow-up durations; **b** subgroup analysis according to the study design characteristics

Eleven datasets from eight follow-up studies evaluated the association between serum 25(OH) D and mortality risk in patients with sepsis [19–26]. Results of Cochrane’s Q test (*p* = 0.003) and the estimation of I^2^ (63%) indicated significant heterogeneity. Meta-analysis with a random-effect model showed that septic patients with lower serum 25 (OH) D (< 30 ng/ml) at admission was associated with higher mortality risk (adjusted RR: 1.93, 95% CI: 1.41 to 2.63, *p* < 0.001; Fig. 2). Sensitivity analysis by excluding one study at a time did not significantly change the results (adjusted RR: 1.76 to 2.11, p all <0.01). Subgroup analyses according to the severity of vitamin D deficiency suggested that patients with severe vitamin D deficiency was significantly associated with higher mortality risk (adjusted RR: 1.92, 95% CI: 1.09 to 2.55, *p* < 0.001; Fig. 3a), but the associations were not significant in patients with vitamin D insufficiency and deficiency (*p* = 0.08 and 0.25 respectively; Fig. 3a). Further analyses showed that the association between lower serum 25(OH) D and higher mortality risk were consistent in studies applied different diagnostic criteria for sepsis (SIRS, Sepsis-2.0, or Sepsis-3.0; Fig. 3b), short-term (within 1 month) and long-term studies (3~12 months; Fig. 4a), and in prospective and retrospective studies (Fig. 4b).

### Publication bias

**Fig. 5:**
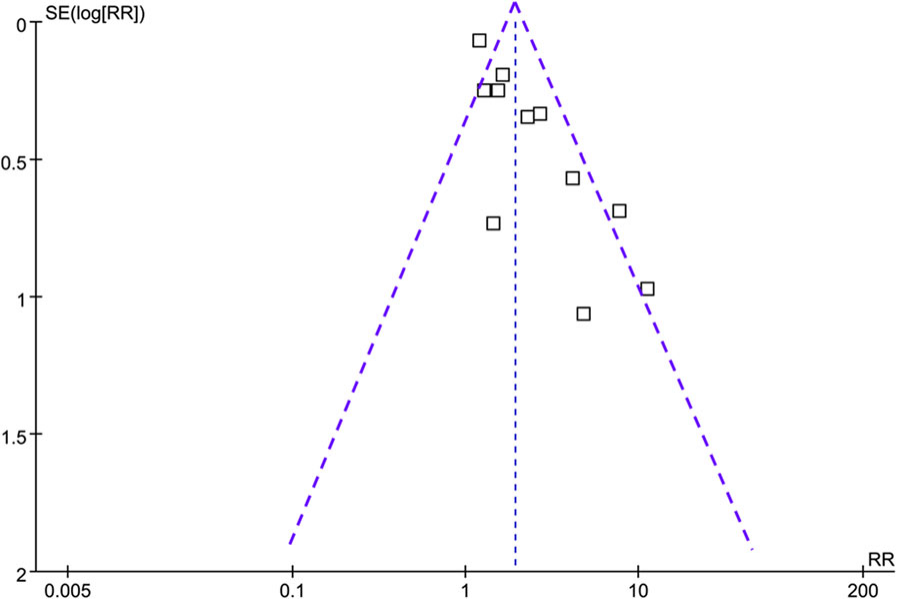
Funnel plots for the meta-analysis of the association between serum 25 (OH) D and mortality risk in patients with sepsis

The funnel plots for the main meta-analysis were shown in Fig. 5, which was symmetrical on visual inspection, indicating low risk of publication bias. Further evaluation by Egger’s regression test also indicated low risk of publication bias (*p* = 0.211).

## Discussion

In this meta-analysis of multivariate adjusted follow-up studies, we found that vitamin D deficiency as evidenced by lower serum 25 (OH) D at admission is independently associated with increased mortality for adult patients with sepsis. Further subgroup analyses showed that the association between lower serum 25 (OH) D and higher mortality risk in these patients were mainly retrieved by datasets with severe vitamin D deficiency (25 (OH) D < 10 ng/ml). Moreover, the association between lower serum 25 (OH) D and higher mortality risk were consistent in studies with different diagnostic criteria for sepsis (SIRS, Sepsis-2.0, or Sepsis-3.0), short-term (within 1 month) and long-term follow-up studies (3~12 months), and in prospective and retrospective studies. Taken together, these results indicated that severe vitamin D deficiency may be independently associated with increased mortality in patients with sepsis. Large-scale prospective studies are needed to validate our findings.

Although vitamin D deficiency has been confirmed to be an independent predictor of poor prognosis in patients with critical illness in previous studies [15, 16, 18], previous studies included patients with heterogeneous critical illnesses and a subgroup analysis in patients with sepsis was rarely performed. Our meta-analysis, focused on adult patients with sepsis, showed that lower serum 25 (OH) D is significantly associate with higher mortality in these patients. A previous meta-analysis with five studies failed to show a significant association between vitamin D deficiency and poor prognosis in patients with sepsis [17]. By incorporating up-to-date clinical studies, our meta-analysis demonstrated that lower serum 25 (OH) D is independently associated with increased mortality in adult patients with sepsis. It should be noticed that this finding is based on data after adjustment of current risk stratification scores for patients with critical illness. Therefore, our results highly indicated that vitamin D deficiency may be an additional independent predictor of mortality risk in patients with sepsis. Moreover, we performed sensitivity and subgroup analyses to evaluate the stability of the findings, which showed that the result was consistent regardless of the follow-up durations and designs of the study. Interestingly, our pilot subgroup analyses showed that the significance of the association between lower serum 25 (OH) D and higher mortality in patients with sepsis were mainly driven by studies with severe vitamin D deficiency, rather than studies with vitamin D deficiency or insufficiency. Despite of its conventional role as a nutrient, vitamin D has been demonstrated to play important roles in many physiological processes involved in the pathogenesis of sepsis [34]. Currently, sepsis is defined as life threatening organ dysfunction caused by dysregulated host response to infection [35]. Sufficient vitamin D is important for maintaining and regulating both the innate and adaptive immune system, which may therefore exert protective effect against severe infection and overactivated inflammatory response [36]. Moreover, via interaction with widely distributed vitamin D receptor and subsequent signaling pathways, vitamin D could maintain the functional statuses of multiple organs which are vulnerable during severe infection, such as heart [37], lung [38], and kidney [39] etc. Future studies are needed to determine the key molecular pathways underlying the association between vitamin D deficiency and poor prognosis in patients with sepsis.

Results of our study further highlighted the hypothesis that supplementation of vitamin D may reduce the mortality in patients with sepsis and severe vitamin D deficiency. Randomized controlled trials (RCTs) are rarely performed to evaluate the role of vitamin D supplementation on clinical outcomes in patients with sepsis and vitamin D deficiency. One of the included study of our meta-analysis which enrolled 57 ICU patients with sepsis did not show that exogenous vitamin D3 supplementation was associated with improved 28-day accumulated survived rate [23]. A previous RCT including 475 ICU patients with vitamin D deficiency (25 (OH) D < 20 ng/ml) failed to show the benefit of vitamin D supplementation on mortality in overall population [40], but subsequent subgroup analysis showed that vitamin D supplementation significantly improved survival in patients with severe vitamin D deficiency (25 (OH) D < 12 ng/ml). Interestingly, results of subgroup analyses also indicated that only septic patients with severe vitamin D deficiency were associated with higher mortality, but not for those with vitamin D deficiency or insufficiency. Taken together, these results may indicate that vitamin D supplementation could improve survival in sepsis patients with severe vitamin D deficiency. However, only about 800 patients were included in current RCTs evaluating the clinical benefit of vitamin D supplementation in patients with critical illnesses, and none of them were focused on patients with severe vitamin D deficiency [41–43]. Clinical trials with adequate sample size and including patients with severe vitamin D deficiency are needed to evaluate the potential benefits of vitamin D on survival in patients with critical illnesses, including sepsis.

Our study has some limitations. Firstly, the number of the included studies in the meta-analysis is limited, which prevented us from further investigating into the potential influences of study characteristics on the outcome, such as the nutritional status, morbidities, and concurrent therapies of the patients. Secondly, the cut-off values for defining vitamin D deficiency varied in the included studies. Future studies are needed to determine the optimal cut-off value of vitamin D deficiency that confers the predictive efficacy for overall mortality in patients with sepsis. Thirdly, although we combined the results of the most adequately adjusted data from the included studies, it remains possible that some residual factors may confound the association between lower serum 25 (OH) D and mortality risk. Fourthly, a causative relationship between vitamin D deficiency and poor survival could not be derived from our study since it is a meta-analysis of observational studies. Fifthly, immunoassay was applied for the measuring of serum 25 (OH) D in the included studies rather than high-performance liquid chromatography (HPLC), liquid chromatography–mass spectrometry (LC/MS), or liquid chromatography-tandem mass spectrometry (LC-MS/MS) [44, 45]. Among these measuring methods for 25(OH) D, LC-MS/MS is considered as the “gold standard” method because of its high sensitivity, specificity, and accuracy [46]. The influence of different methods for the measurement of serum 25(OH) D on the outcomes should also be evaluated in future studies. Finally, a previous study showed that lower 1,25-dihydroxyvitamin D (1,25 (OH)_2_D), the active metabolite of 25(OH) D, is associated with lower risk of mortality in patients with sepsis [47]. Whether the independent association between lower 25(OH) D and poor prognosis in septic patients is mainly mediated by reduced 1,25 (OH)_2_D deserves further investigation.

## Conclusions

In conclusion, results of our meta-analysis indicated that severe vitamin D deficiency may be independently associated with increased mortality risk in adult patients with sepsis. Large-scale prospective studies are needed to validate our findings.

## Supplementary information

**Additional file 1.** Search strategy for PubMed.

## Data Availability

All relevant data for this study are presented in tables, figures and supplementary materials.

## Abbreviations

25 (OH) D: 25-hydroxyvitamin D
CI: Confidence intervals
ED: Emergency department
ICU: Intensive care unit
LC-MS/MS: Liquid chromatography-tandem mass spectrometry
MOOSE: Meta-analysis of Observational Studies in Epidemiology
NOS: Newcastle-Ottawa Scale
RR: Relative risk
SEs: stand errors
SIRS: Systemic inflammatory response syndrome
