# Supplementary material for: Serum 25-Hydroxyvitamin D and the risk of mortality in adult patients with Sepsis: a meta-analysis
Source: BMC Infect Dis. 2020 Mar 4;20:189. doi: 10.1186/s12879-020-4879-1 (PMC7057612; doi:10.1186/s12879-020-4879-1)
Supplement: Supplementary file 1 — Additional file 1. Search strategy for PubMed. [file 12879_2020_4879_MOESM1_ESM.docx]

**Search strategy for PubMed**

((((((Sepsis) OR septicemia) OR septic)) OR septicem*or septicaem*or seps*)) AND (((((vitamin d) OR ((((((((((("vitamin d"[MeSH Terms]) OR 25-hydroxyvitamin D) OR 1,25-dihydroxyvitamin D) OR 1,25(OH)(2)D) OR Vitamin D2) OR Vitamin D3) OR Ergocalciferol) OR Cholecalciferol) OR Calcidiol) OR calcifediol) OR Calcitriol)))))
